# Prognostic factors of survival time after hematopoietic stem cell transplant in acute lymphoblastic leukemia patients: Cox proportional hazard versus accelerated failure time models

**DOI:** 10.1186/1756-9966-27-74

**Published:** 2008-11-23

**Authors:** Kourosh Sayehmiri, Mohammad R Eshraghian, Kazem Mohammad, Kamran Alimoghaddam, Abbas Rahimi Foroushani, Hojjat Zeraati, Banafsheh Golestan, Ardeshir Ghavamzadeh

**Affiliations:** 1 Department of Biostatistics, School of Public Health, Tehran University of Medical Sciences, Iran; 2 Hematology-Oncology and Stem Cell Transplantation Research Center, Tehran University of Medical Sciences, Iran

## Abstract

**Background:**

The aim of this study is to evaluate the prognostic factors of overall survival (OS) after haematopoietic stem cell transplant (HSCT) in acute lymphoblastic leukaemia (ALL) patients using accelerated failure time (AFT), Cox proportional hazard (PH), and Cox time-varying coefficient models.

**Methods:**

206 patients were enrolled after HSCH in Shariati Hospital between 1993 and 2007. There was evidence of marked departures from the proportional hazards assumption with two prognostic factors, relapse and chronic graft-versus-host disease (cGVHD) (P < .001). Performance among AFT and Cox's models was assessed using explained variation and goodness of fit methods. Discrimination among the exponential, Weibull, generalized gamma (GG), log-logistic, and lognormal distributions was done using maximum likelihood and Akaike information criteria.

**Results:**

The 5-year OS was 52% (95%CI: 47.3–56.7). Peak mortality hazard occurred at months 6–7 after HSCT followed by a decreasing trend. In univariate analysis, the data was better fitted by GG distribution than by other distributions. Univariate analysis using GG distribution showed a positive association between OS with acute graft-versus-host disease (aGVHD) (P = .021), no relapse (P < .001), cGVHD (P < .001), neutrophil recovery (P < .001) and platelet recovery (P < .001). Based on Cox PH models; however cGVHD and relapse were the predictive factors of OS (P < .001). Multivariate analysis indicated that, OS is related to relapse (P < .001) and platelet recovery (P = .037), where predictive power of Weibull AFT models was superior to Cox PH model and Cox with time-varying coefficient (R^2 ^= 0.46 for AFT, R^2 ^= .21 for Cox PH and R^2 ^= .34 for Cox time-varying coefficient). Cox-Snell residual shows Weibull AFT fitted to data better than other distributions in multivariate analysis.

**Conclusion:**

We concluded that AFT distributions can be a useful tool for recognizing prognostic factors of OS in acute lymphoblastic leukemia patients.

## Background

Identifying prognostic factors of patients' survival time after Hematopoietic stem cell transplant (HSCT) is of importance not only because it enables the physicians to detect the factors whose changes affect patients' survival time, but also helps them to make the best decision about patients' treatment.

Several factors are known to predict long-term survival of acute lymphoblastic leukemia patients, including age, white blood cells count, lactic dehydrogenate level, karyotype, stage of the disease at the time of the transplant, Cyclosporin for preventing graft-versus-host disease and donor-recipient sex combination[1,2]. The role of some of this prognostic factors such as acute graft-versus-host disease(aGVHD), chronic graft-versus-host disease(cGVHD), age, sex are controversial [2-6], for instance aGVHD was reported a significant factor for survival after HSCT [4,6], whereas it was not a predictive factor in other studies[5,7]. These differences may be due to a methodological issue.

Prognostic factors of acute leukemia after HSCT are already identified by using nonparametric survival methods such as Kaplan-Meier and Cox Proportional Hazard (PH) in many studies[1,8-13]; the latter is used when the effect of covariates on the hazard ratio is desired. Review of literature shows the extensive use of the Cox PH regression model for hazard rate or instantaneous risk of a given event [14,15]; however, the basic and the most important assumption underlying this model is proportionality of hazard rates, which may not be held in some situations. Where PH assumption is not met, it is improper to use standard Cox PH model as it may entail serious bias and loss of power when estimating or making inference about the effect of a given prognostic factor on mortality [15-18]. A review of survival analysis in cancer journals reveals that only 5% of all studies using the Cox PH model considered the underline assumption[14].

Recently, AFT models as parametric models have attracted considerable attention, because not only they do not need PH assumption but also thanks to availability of standard methods such as Maximum Likelihood (ML), parameter estimation and testing can be done readily[18].

When survival time has a specific statistical distribution, the statistical power of parametric survival models is higher than nonparametric or semi-parametric survival models. The exponential, Weibull, log-logistic, lognormal and the generalized gamma (GG) are among parametric distributions commonly used for studying survival time analysis. Survival estimates obtained from parametric survival models typically yield plots that are more consistent with a theoretical survival curve [17].

Like Cox PH model, parametric survival models can be used in regression forms. The interpretations of parameters for AFT models are also different from Cox PH models. The AFT assumption is applicable for a comparison of survival time whereas the PH assumption is applicable for the comparison of hazards[18,19].

Since recently AFT models have not been used very often and the few usage of these models are found in kidney transplant studies[20,21], based on our knowledge, it has not been used to recognize the prognostic factors of acute leukemia patients so far. In this Paper, we tried fitting AFT models, chose the one with the best fitness and used it to determine prognostic factors for survival after HSCT in acute leukemia patients. We did also compare the results of AFT models with Cox PH and Cox time-varying coefficients models.

## Methods

### Data Collection and Patient Selection

Data on patients who underwent bone marrow or peripheral-blood transportation from HLL identical siblings were obtained from the Hematology-Oncology and Stem Cell Transplantation Research Center at Shariati Hospital, Tehran, Iran. Transplantations were performed between Oct 17, 1993 and Jan 31, 2007.

All patients received a BuCy regimen (busulfan 4 mg/kg/day orally on days-6 to-3 and cyclophosphamide 60 mg/kg/day by intravenous infusion on days-2 to -1) for conditioning therapy with subsequent infusion of donor marrow cells on day 0. For graft-versus-host disease (GVHD) prophylaxis all patients received conventional Protocol Cyclosporin 3 mg/kg/day IV from days-2 and methotrexate 10 mg/m^2 ^day +1 and 6 mg/m^2 ^on days 3, 6 and 11. We changed Cyclosporin to oral formulation when oral intake was possible.

All patients' records were reviewed for the occurrence of adverse events including GVHD and regimen-related toxicities. There were 206 patients eligible for this longitudinal study. Patients in this study were in the age range of 2–56 years old and had received a HLA-matched marrow transplant. The median follow up time after transplantation was about 1.5 years.

### Definition of Endpoints

Platelet recovery was defined by a count of at least 20,000 platelets per micro liters, unsupported by transfusion for seven days.

#### Hematopoietic recovery

Neutrophil recovery was defined by an absolute neutrophil count of at least 500 cells per cubic millimeter in three consecutive days.

#### GVHD

The incidence of acute GVHD (aGVHD) was determined in all patients. Acute GVHD was graded according to the Seattle criteria [13,22]. The aGVHD grade of 1,2,3,4 was defined for having aGVHD. Chronic GVHD (cGVHD) was defined according to standard criteria [13,22]. The incidence of chronic GVHD (cGVHD) was determined in patients who survived for at least 90 days [13,23,24].

#### Relapse

Relapse was defined as a recurrence of leukemia confirmed by cytology.

#### Survival

Overall Survival (OS) was defined as the time interval between HSCT and death of any cause related to acute leukemia or censoring. Censoring was defined as being alive at the last follow-up. According with the specific goals of the analysis, we did not classify the events (deaths) according to theirs reasons.

#### Time ratios (TR)

AFT model coefficients are most intuitively expressed in the exponential form, a TR > 1 associates with a prolonged survival time whereas a TR < 1 is associated with a decrease in survival time[25].

### Statistical Analysis

The probability of OS was estimated using Kaplan-Meier estimator. Confidence intervals were calculated via Log transformation[18]. The quantile-quantile(QQ) plot was used to check the adequacy of AFT assumption[18].

AFT models such as the exponential Weibull, Log-Logistic, lognormal and Generalizes Gamma (GG) distributions were used for finding the best distribution fitted to time to event after HSCT. To find the best fitted model among GG family distributions such as; the Exponential, Weibull, lognormal and GG we used maximum likelihood (ML) and Akaike information criteria(AIC) as well as using graphical methods, namely Cox-Snell residuals [16-19]. Discrimination among distributions of the GG family was done using likelihood-ratio chi-square test[19]. Akaike information criteria was use to compare the best fitted model in the GG family with log-logistic distribution.

Adequacy of the AFT models was gauged by a liner function of cumulative hazard rate versus appropriate function of survival time: for exponential, a plot of -log*Ŝ*(t) versus t, for Weibull, a plot of log [-log*Ŝ*(t)] versus log t, for log-logistic, a plot of log [(1-*Ŝ*(t))/*Ŝ*(t)] versus log t [16,18,19].

Deviance residuals and Martingale residuals were considered for checking outliers and influential observations in models[18,19,26].

Model performance among AFT and Cox's was achieved using the explained variation and goodness of fit test(GOF) methods[16,27]. Conditional distributions of parametric (AFT models) survival time models were estimated by including different covariates in the models.

AFT models were used for finding prognostic factors of survival after HSCT. PH assumption was checked using graphical methods(log cumulative hazard rate versus survival time), score residuals, the scaled Schoenfeld residuals and time-depended variable procedures [18,19,28].

Smoothed hazard function was estimated using Kernel smoothing method (Epanechnikov function)[16,29]. P-value less than .05 was considered significant. Analyses were done using STATA ver. 8.

## Results

### General Description

**Figure 1:**
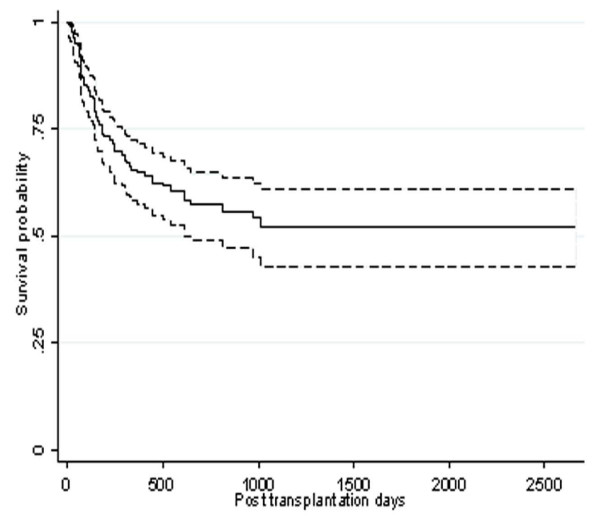
Kaplan-Meir estimated survival after transportation for patients diagnosed with acute lymphoblastic leukemia.

One hundred and thirty nine (67.5%) of patients and eighty five (41.3%) of donors were female. Table 1 shows the characteristics of 206 patients who were included in the study. Based on Kaplan-Meier curve, the 5-year-survival rate was 52 % (95% CI: 47.3–56.7) (Figure. 1), for patients in the first complete remission (CR1) this rate was estimated as 65% (CI 95%: 60.1–69.9).

**Table 1:** Patients and Transplants Characteristic

**Characteristic**	**Frequency (%)**
**D**onor-recipient sex match-no. (%)	
Male-male	85(41.3)
Male-female	54(26.2)
Female-male	36(17.5)
Female-female	31(15)
**A**ge Mean(SD)	22.5(8.73)
**A**ge(years), Median(range)	20(2–51)
**D**onor age, Median(range)	21(2–55)
**A**ge group – no. (%)	
< 15 yr	37(18)
16–20 yr	72(35)
21–30 yr	64(31.1)
31–40 yr	22(10.7)
> 40 yr	11(5.3)
**O**utcomes-no. (%)	
Death	76(39.9)
Relapse	59(28.6)
aGVHD	136(77.7)
cGVHD	34(24.1)
Platelet recovery	141(75.0)
Neutrophil recovery	167(85.6)
**T**ime of aGVHD-day	
Mean(SD)	13.3(16.5)
Median	9
Range	3–90
**T**ime of cGVHD-day	
Mean(SD)	160.26(73.4)
Median	140
Range	91–327
**T**ime of relapse-day	
Mean(SD)	580(555)
Median	412
Range	10–2661
**T**ime of platelet recovery-day	
Mean(SD)	20.8(19.2)
Median	17
Range	1–165
**T**ime of neutrophil recovery-day	
Mean(SD)	13.46(12.8)
Median	11
Range	1–160
	
**F**ollow up-month	
Median	16
range	3–89

aGVHD, acute graft-versus-host disease; cGVHD, chronic graft-versus-host disease

**Figure 2:**
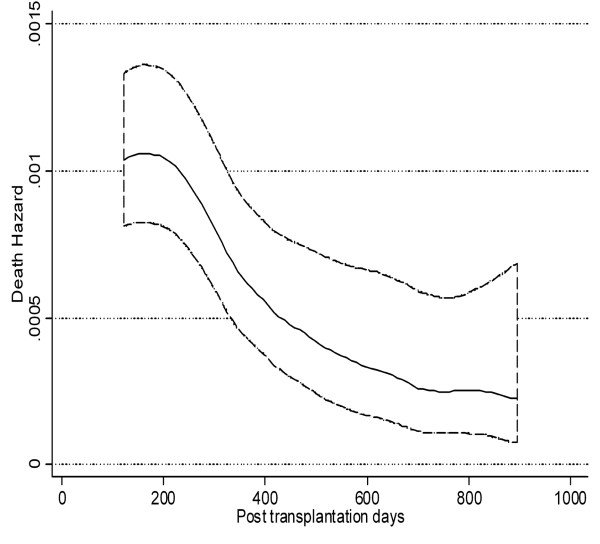
Smoothed death hazard in acute lymphoblastic leukemia patients after transplantation.

The shape of hazard function for mortality revealed a peak at 6–7 months after HSCT followed by a decreasing trend as hazard of dying in the first 6 months after transplantation was higher than the second six months (Figure 2). The shape of hazard function in Figure 2 suggests the appropriateness of the generalized gamma, log normal or log-logistic distributions. The gamma and log normal are more preferable when hazard rises to a peak before decreasing.

In the absence of covariates, GG is the best fitted mode Among AFT models (it has the smallest AIC) (table 2); therefore the values of goodness-of-fit other distributions were compared to GG distribution.

**Table 2:** Discrimination among Distributions Using Maximum Likelihood (LL) and Akaike Information Criteria (AIC) (n = 206)

			**GG family**					
					
Model No.	Variables name	Goodness of fit criteria	Exponential	Weibull	Log normal	GG	Log-logistic	Best model
1	Without covariate	-LL AIC	251.7 503.4	240.9 483.8	233.1 468.2	227.3 458.6	236.6 475.2	GG
2	aGVHD	-LL AIC	231.1 464.2	218.6 441.2	211.1 426.2	202.6 411.2	214.7 423.4	GG
3	cGVHD	-LL AIC	225.7 453.4	212.5 429.0	204.4 412.8	195.1 396.2	208.1 420.2	GG
4	Platelet recovery	-LL AIC	240.6 438.2	231.0 466.0	223.2 450.4	214.4 434.8	227.2 458.4	GG
5	Relapse	-LL AIC	216.2 434.4	213.2 430.4	219.3 442.6	210.5 427.0	218.0 440.0	GG
6	Neutrophil recovery	-LL AIC	248.5 499.0	238.2 480.4	230.2 464.4	218.9 443.8	234.2 472.4	GG
7*	aGVHD, cGVHD, relapse, platelet recovery, neutrophil recovery	-LL AIC	181.2 372.4	177.9 **367.8**	179.1 370.2	178.1 370.2	179.1 370.2	Weibull
8	aGVHD, cGVHD, relapse, platelet recovery, neutrophil recovery patients' age and sex	-LL AIC	180.4 374.8	177.2 370.4	177.6 371.2	177.4 372.8	178.0 372.0	Weibull

*Final Model, GG; Generalized Gamma, cGVHD; chronic graft-versus-host disease. AIC = -2LL+2P, where p is the number of parameters in the model, Model number 7 is the best fitted model because it has the smallest AIC.

Since Exponential, Weibull and log-normal distributions are nested in GG distribution, the difference between Log-likelihood (LL) of GG model and its nested model multiplying by 2 yields the following likelihood-ratio chi-square statistics:

X^2 ^= 48.8, df = 2, P = < 0.001, Exponential vs. GG

X^2 ^= 27.2, df = 1, P = < 0.001, Weibull vs. GG

X^2 ^= 5.8, df = 1, P = .016, Lognormal vs. GG

Likelihood-ratio chi-square statistics and AIC show GG fits the data better than exponential, Weibull, log-normal distributions. Moreover in the absence of covariates, AIC showed that GG model was a better fit than log-logistic distribution (Table 2).

### Prognostic Factors of Survival after HSCT, Univariate Analysis

**Figure 3:**
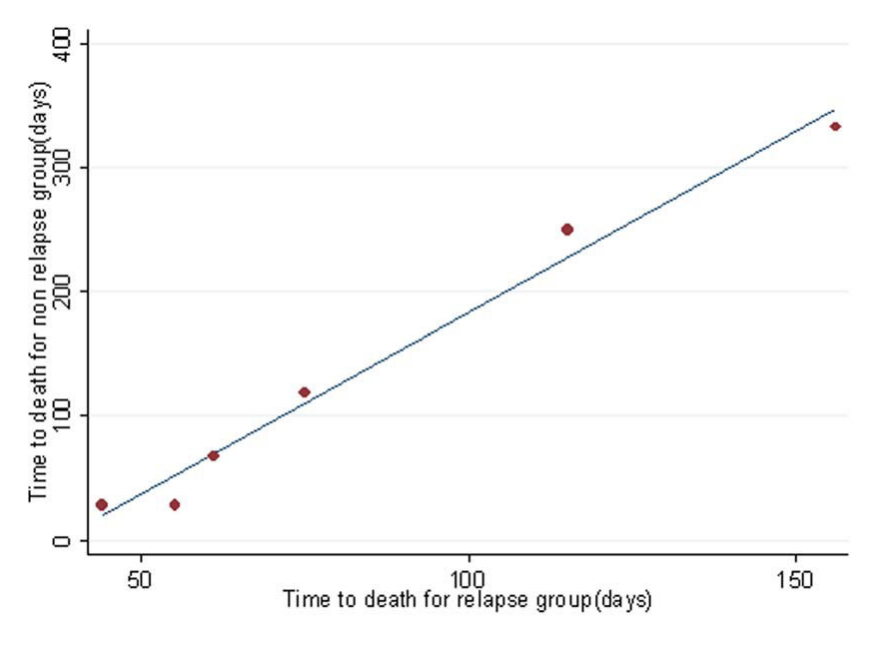
QQ plot for time to death of acute lymphoblastic leukemia patients after transplantation grouped according to relapse development.

**Figure 4:**
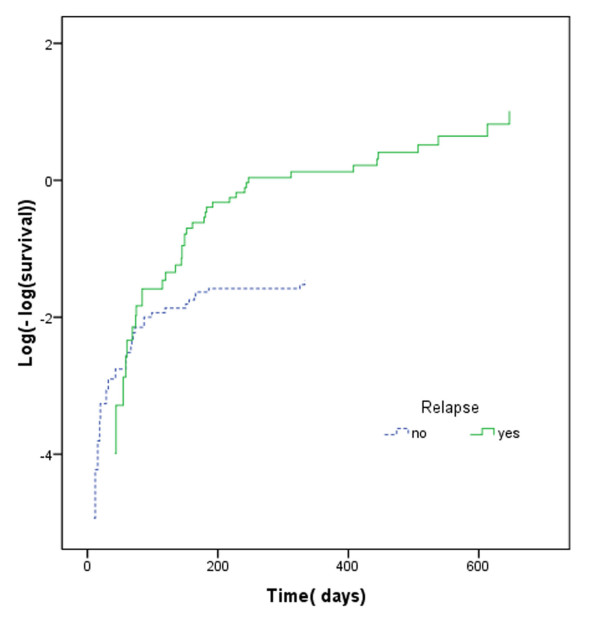
Log(-log(survival) curve for time to death of acute lymphoblastic leukemia patients after transplantation grouped according to relapse development.

Quantile-quantile(QQ) plots provide the adequacy of AFT models in univariate analysis. We show QQ plot for relapse in figure 3. Maximum likelihood (ML), AIC and graphical methods all showed that, in univariate analysis, the GG model fitted the data better than other AFT models (Table 2). Proceeding with GG model, univariate analysis showed that there is a significant association between OS and relapse, aGVHD, cGVHD, neutrophil recovery and platelet recovery (Table 3), whilst Cox PH revealed a significant association between OS and both relapse and platelet recovery (Table 3). Table 3 shows that there is a strong correlation between OS and leukemia recurrence after transplantation (P < .001, Time Ratios (TR) = 10). Median OS in patients who have had relapse after transplantation was about 10 times shorter than others. The assumption of proportionality was not met for relapse in the Cox PH model (P = < .0001) (Figure 4). Based on our results, the hazard of death in patients with recurred relapse is 4.8 times higher than other patients (Table 3). The constancy of hazard ratio over time is not unrealistic, given that PH assumption is not met for the model (Figure 4).

**Table 3:** Prognostic Factors of OS in Univariate Analysis Using Generalized Gamma and Cox Model (n = 206).

	Generalized Gamma		Cox PH			
Characteristics	TR (95% CI)	p-value	HR (95% CI)	p-value	proportional assumption	p-value
Relapse(yes vs. no)	10(3.2–21.27)	< .001	4.79(2.99 7.67)	< .001	Not met	< .001
aGVHD(yes vs. no)	2.29(1.13–4.71)	.021	.82(.46 2.03)	.49	Met	.68
cGVHD(yes vs. no)	5(2.27–10.71)	< .001	.53(.27 1.05)	.072	Not Met	.004
Platelet recovery(yes vs. no)	3.39(1.19–6.1)	< .001	.51(.31 .82)	.006	Met	.48
Neutrophil recovery(yes vs. no)	3.60(1.90–6.9)	< .001	.65(.35 1.21)	.18	Met	.11

aGVHD; acute graft-versus-host disease, cGVHD; chronic graft-versus-host disease, PH; proportional hazard, HR; hazard ratio, TR; time ratio

Note: the corresponding regression coefficients in the above models can be obtained by taking logarithm of time ratio and hazard ratio.

**Figure 5:**
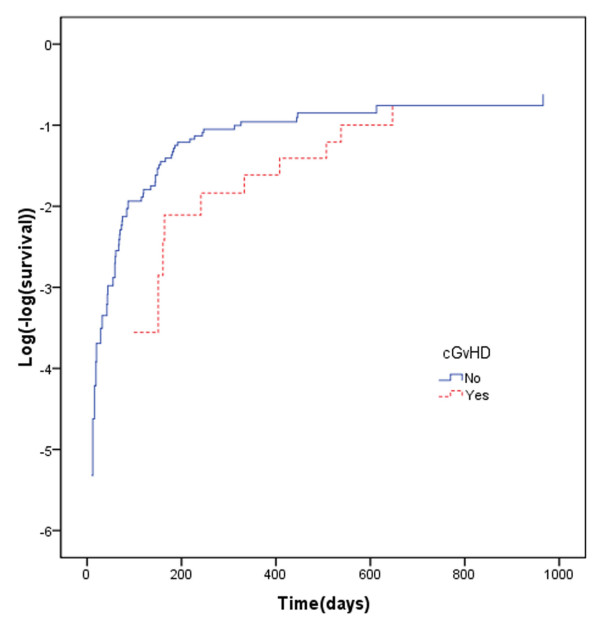
Log(-log(survival) curve for time to death of acute lymphoblastic leukemia patients after transplantation grouped according to cGVHD development.

Incidence of cGVHD, among patients who survived for 90 days or longer after transplantation was 24.1%. For these patients there was a significant association between cGVHD and OS (P < .001 TR = 5), indicating that median OS was about 5 times longer in the acute leukemia patients with cGVHD compare to those without it (table 3); However since the assumption of proportionality was not met for cGVHD in the Cox PH model (P = < .003, Figure 5), the interpretation of HR may be questionable.

### Prognostic Factors of Survival after HSCT, Multivariate Analysis

Variables with significance level less than 0.2 in univariate analysis were considered in the multivariate models. Based on AIC criteria (with the smallest AIC as the best), the model including relapse, platelet recovery, neutrophil recovery, aGVHD and cGVHD is the best for prediction of OS (final model in table 2).

**Figure 6:**
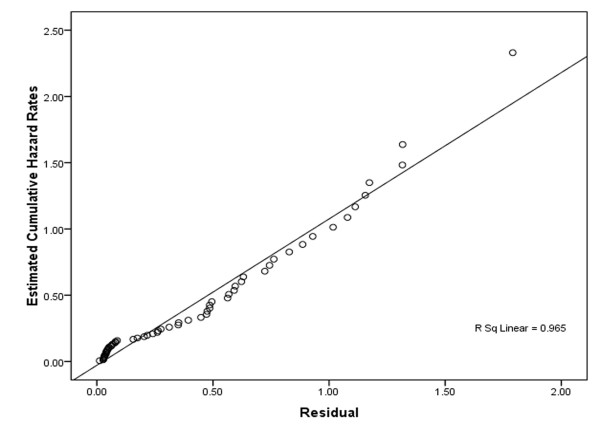
Cox-Snell residuals to assess the fit of the Weibull model for time to death of acute lymphoblastic leukemia patients after transplantation.

Weibull distribution has the smallest AIC; therefore it is the best fitted model on data. Goodness-of-fit of exponential distribution versus Weibull distribution must be rejected(X^2 ^= 6.6, df = 1, P = .01). Figure 6 shows that, plot of Cox-Snell residuals versus the Nelson-Aalen estimator or the cumulative hazard of the residuals is straight line with slop one indicating adequacy of the fitted Weibull distribution.

Nevertheless, since PH assumption was not met for this model (test for scaled Schoenfeld residuals; for relapse: X^2 ^= 26.91 P = .000, for cGVHD: X^2 ^= 8.28, P = .004, global test: X^2 ^= 42.5, df = 5 P < .001) the interpretation of hazard ratios (HRs) of Cox model in Table 4 may be questionable. When PH assumption is not met for a model, we can usually use AFT or Cox model with time-varying covariates. Explained variation, AIC criteria and graphical methods (Figure 6) show that the Weibull AFT is the best model comparing with Cox PH and Cox with time-varying coefficients. Table 4 shows interaction between time and both cGVHD and relapse in a Cox model with time-varying coefficients, so their hazard ratios cannot be interpreted separately.

**Table 4:** Prognostic Factors of OS in Multivariate Analysis Using AFT and Cox's Models (n = 206).

	Weibull AFT		Cox PH		Cox with time-varying coefficients	
Characteristics	Time ratio(95% CI)	p-value	HR (95% CI)	p-value	HR (95% CI)	p-value
Relapse(yes vs. no)	.082(.039 .17)	< .001	5.2(3.1 8.94)	< .001	.69(.25 1.8)	.062
aGVHD(yes vs. no)	.95(.45 2.03)	.91	1.09(.61 1.95)	.76	.96(.53 1.74)	.90
cGVHD(yes vs. no)	1.52(.62 3.72)	.35	.71(.35 1.43)	.34	.16(.04 .62)	.008
Platelet recovery(yes vs. no)	2.37(1.05 5.32)	.037	.52(.28 .96)	.039	.53(.28 1.03)	062
Neutrophil recovery(yes vs. no)	2.71(.62 11.85)	.184	.46(.14 1.44)	.187	.39(.12 1.24)	.12
						
T_i _*cGVHD					1.006(1.002 1.011)	.003
T_i _*relapse					1.012(1.006 1.019)	< .001
						
Explained variation(R^2^)	.46		.21		.34	

aGVHD; acute graft-versus-host disease, cGVHD; chronic graft-versus-host disease, AFT; accelerated failure time, PH; proportional hazard, HR; hazard ratio, T_i_; survival time.

Note: the corresponding regression coefficients in the above models can be obtained by taking logarithm of time ratio and hazard ratio.

## Discussion

Our objective was to identify prognostic factors of OS using parametric (AFT models) and semi-parametric methods (Cox's models). Peak mortality hazard occurred at months 6–7 after transplantation with a decreasing rate afterwards. In a model without any covariate and in univariate analysis GG distribution fitted the data better than other parametric survival models such as the exponential, Weibull, log-normal, log-logistic distributions (table 2). It has been shown that hazard function in the GG distribution can take a wide variety of shapes [16], one of which is when it reaches a maximum and then decreases [30]. To our knowledge, no other study of this type has ever worked on prognostic factors of OS in ALL patients using GG distribution.

In many researches, Cox PH regression models were used to consider prognostic factors of OS in acute leukemia patients [11,12,21,24,31,32]. The results of this research show that Cox PH models may fail to identify prognosis factors of OS in acute lymphoblastic leukemia patients. As our results indicated, in univariate analysis, neutrophil recovery, platelet recovery and aGVHD were significant prognostic factors of OS using GG distribution whereas they failed to be significant prognostic factors when using Cox PH model (Table 3). However, even if the assumption of PH holds, we may still get different results with these two models. It seems that patients, who have neutrophil recovery, survive longer comparing to the patients without it. Based on our findings, the result of GG distribution makes more sense and looks more reasonable than Cox PH model.

For the final model of the multivariate analysis, the hazard ratio for covariates may not have a clear interpretation as the assumption of PH was not held Here clearly using AFT survival models is advantageous over Cox PH model as it does not require the assumption of PH (9). Moreover, AFT models not only can specify a direct relation between the logarithm of survival time and a set of explanatory variables, but also permits a clearer interpretation of the effect of each covariate on survival, allowing to estimate the median event times.

Maximum likelihood, AIC (table 1), and Cox-Snell residual plots (Figure 6 show Weibull AFT is the best fitted model among AFT models. There are two types of Weibull model: Weibull AFT and Weibull PH; but only for the latter is the assumption of PH indispensable; thereby when it does hold we can use either Weibull AFT, Weibull PH or Cox PH[16,17]. In Table 4, explained variations show that predictive power of Weibull AFT is higher than Cox PH and the Cox model with time-varying coefficients.

Usually when PH assumption dose not hold, alternatives are stratified Cox, Cox with time-varying coefficients, AFT models, additive hazard models, and proportional odds models[18,33]. Since PH assumption was not met for the two covariates cGVHD and relapse, following stratification on these covariates not only decreases the power of the analysis (due to small sample size within strata) but also prevents the estimation of effects of the stratified variables. Likewise log-logistic model, the one with the characteristics of both AFT and proportional odds models[18] did not show reasonable fit to our data suggesting proportional odds models are not suitable.

When intermediate events have effect on survival time it is suggested to use the multistate models to describe disease progress. AFT and Cox PH models are among those that can be implemented in such situations [34,35].

It is worth noting that, choosing appropriate model for finding prognostic factors of survival is not an easy task, as checking the goodness of fit could be quite time consuming. Fortunately, there are many methods for checking the goodness of fit of the models[14,16,19,30,36]. We checked our models using some of these methods. These methods could helpfully guide us to come up with the best model for analysis of data.

In our study, based on Kaplan-Meier curves, five-year survival rate in ALL patients at CR1 disease stage was estimated to be 65% (CI 95%: 60.1–69.9). The Center of International Blood and Marrow Transplant Research (CIBMTR) and the National Marrow Donor Program (NMPD) has reported 65% survival rates in ALL patients [37], showing that the patients of this study may be a good candidate of all acute lymphoblastic leukemia patients.

The multivariate analysis using the Weibull AFT and Cox models show that relapse and cGVHD are two independent prognostic factors, with the adverse effect of relapse on patients OS.

Results showed that, cGVHD developed in 24.1% of ALL patients. In adults, the reported incidence of cGVHD was ranging between 30% and 50% of HLA-identical sibling transplant recipients[38]. In our study developed cGVHD had a positive significant effect on prognosis of ALL patients; Moreover there are some studies reporting the effect of cGVHD on OS [39,40].

## Conclusion

In summary, the results of the current study suggest that when implementing survival analysis in cancer research centers, using the PH model may not be the optimum approach. It is important to identify the distribution of OS and to seek for an appropriate model like AFT models for data analysis. The results from an AFT model are easily interpreted provide a more appropriate description of survival time in many researches, and should be considered as an alternative to the Cox PH model.

The choice of the appropriate model will certainly lead to identify more reliable and precise prognostic factors and thereby help to have a more effective treatment program.

## Competing interests

The authors declare that they have no competing interests.

## Authors' contributions

AG and KA participated in the design and acquisition of data, KS performed statistical analysis (Cox regression and AFT models and prepared manuscript), MRE performed PH assumption KZ performed AFT assumption and participate in design study, HZ performed Kernel smoothing for hazard function, ARF help to draft the manuscript, BG carried out explain variation. All authors have read and approved the final manuscript.
